# Comparison of Glycemic and Anthropometric Parameters Among Patients With Dyspepsia and Dyslipidemia With or Without Helicobacter pylori Infection: A Cross-Sectional Study

**DOI:** 10.7759/cureus.94811

**Published:** 2025-10-17

**Authors:** Maria Aparecida A Serra, Mateus D Torres, Pedro R Rolins Neto, Marcus Vinicius P de Sousa, Carlos Alberto A Serra dos Santos, Livia M Pascoal, Marcelino S Neto, Leonardo H dos Santos, Marcio Flavio M de Araujo

**Affiliations:** 1 Postgraduate Program in Health and Technology, Federal University of Maranhao, Imperatriz, BRA; 2 Postgraduate Program in Materials Science, Federal University of Maranhao, Imperatriz, BRA; 3 Public Health Research Center, Oswaldo Cruz Foundation, Redenção, BRA

**Keywords:** dyslipidemia, dyspepsia helicobacter pylori, helicobacter pylori, insulin resistance indexes, triglyceride-glucose index (tyg)

## Abstract

Background/purpose: This study aimed to compare glycemic and anthropometric parameters among patients with dyspepsia and dyslipidemia, with or without *Helicobacter pylori* (*H. pylori*) infection.

Methods: This was a cross-sectional study of 103 patients referred for upper gastrointestinal endoscopy (UGE) at a public service city of Imperatriz, state of Maranhão, in Northeastern Brazil. Data collection included structured interviews to collect socioeconomic information, anthropometric assessments, and laboratory tests. Participants were categorized as follows: Group 1: dyslipidemia; Group 2: *H. pylori* infection without dyslipidemia; Group 3: dyslipidemia and *H. pylori* infection; Group 4: neither (control). Triglyceride-glucose (TyG) index, fasting blood glucose, glycated hemoglobin (HbA1c), and anthropometric parameters were compared across all groups. Statistical comparisons used ANOVA or Kruskal-Wallis with appropriate post-hoc tests; significance threshold p<0.05.

Results: The TyG index differed significantly across groups (median (range)): Group 1: 9.10 (8.56-11.5), Group 2: 8.18 (7.60-8.84), Group 3: 8.85 (8.18-9.59), Group 4: 8.30 (7.32-10.0; p<0.001). Body mass index (BMI; kg/m²) and waist circumference (WC; cm) were highest in Group 3 (BMI median 30.2 kg/m^2^; WC mean 106 ± standard deviation (SD) 18.3 cm) (p=0.007 and p<0.001, respectively). No significant differences were observed for HbA1c or fasting glucose.

Conclusion: Dyslipidemia in dyspeptic patients without *H. pylori* infection was associated with higher TyG indices; coexisting dyslipidemia and *H. pylori* infection were associated with increased BMI and WC. No significant differences were observed for HbA1c or fasting glucose between the groups, although the TyG index varied markedly, reflecting discrete changes in insulin sensitivity. These results support integrated screening for dyslipidemia and *H. pylori* in dyspeptic patients to reduce cardiometabolic risk and gastrointestinal diseases.

## Introduction

Insulin resistance (IR) is characterized by reduced tissue sensitivity to insulin, resulting in hyperglycemia and activation of alternative metabolic pathways. This condition is related to the emergence of metabolic disorders, including inflammation, obesity, dyslipidemia, and endothelial dysfunction, which favor the development of diabetes mellitus and cardiovascular diseases [1]. Recent evidence indicates that these metabolic changes have implications for gastrointestinal health and contribute to the development of dyspepsia, chronic gastric diseases, and an increased risk of gastric cancer [2,3].

IR is associated with a state of low-grade chronic inflammation and intestinal dysbiosis, factors that compromise the gastric mucosal barrier and facilitate the colonization and persistence of infectious agents, such as *Helicobacter pylori* (*H. pylori*). This gram-negative bacterium colonizes the human stomach in approximately 50% of the world’s population and is associated with gastrointestinal diseases and gastric cancer [4]. *H. pylori *infection can contribute to the development of IR by inducing chronic inflammation, releasing pro-inflammatory mediators, increasing the production of reactive oxygen species, altering gastrointestinal hormones, such as ghrelin and leptin, and causing insulin secretion dysfunction [5,6].

However, the relationship between *H. pylori* infection and metabolic disorders appears to be bidirectional. Individuals with type 2 diabetes have higher rates of infection [7,8], possibly because of hyperglycemia, which favors bacterial growth and hinders eradication [9,10]. Patients with obesity have a 46% higher risk of *H. pylori* infection [11]. Dyslipidemia can aggravate oxidative stress and impair endothelial function, creating an environment conducive to bacterial colonization [12]. Thus, the presence of IR, obesity, and dyslipidemia can increase the susceptibility to *H. pylori* colonization, whereas infection aggravates preexisting metabolic dysfunction [5].

Various studies have presented divergent results regarding the effect of *H. pylori* infection on metabolic disorders, especially when analyzing only isolated components of the metabolic syndrome. Although some studies have suggested an association between infection and dyslipidemia [12,13], hyperglycemia [7,8], and high triglyceride-glucose (TyG) indices [14,15], others have indicated that the presence or eradication of bacteria may not influence insulin sensitivity and serum lipid profiles [16-19], besides contributing to increased body mass [11,16]. These heterogeneous findings reflect the complexity of the interactions between metabolic and infectious factors, raising questions about the possible synergistic effects of chronic gastrointestinal infection and metabolic imbalance.

Thus, we hypothesize that patients with dyslipidemia will exhibit greater IR, as estimated by the TyG index, than those with isolated *H. pylori* infection, and that the coexistence of both conditions will result in a more obesogenic anthropometric profile.

Although previous studies have explored associations between *H. pylori *infection and metabolic parameters, results remain heterogeneous, and few have stratified dyspeptic patients simultaneously by the presence of dyslipidemia and infection. To our knowledge, no study has directly compared glycemic and anthropometric parameters across four clinically relevant strata: isolated dyslipidemia, isolated *H. pylori* infection, coexistence of both, and absence of both in a dyspeptic endoscopy population. Therefore, this study aims to address this gap by providing a novel comparative analysis to clarify how the interaction between dyslipidemia and *H. pylori* infection may modulate the metabolic profile of dyspeptic patients.

## Materials and methods

This cross-sectional study was conducted at a public endoscopy service in Imperatriz, Maranhão, Northeastern Brazil. The facility provides gastroenterology care and free examinations for patients hospitalized or referred from primary or secondary healthcare units. This service performs approximately 120 upper gastrointestinal endoscopies (UGEs) per month. The sample size was estimated to include 92 patients, assuming Z = 1.96 and a type I error of 5%, according to the following formula:

$n = \frac{N Z^2 p (1 - p)}{Z^2 p (1 - p) + e^2 (N - 1)}$

where n is the calculated sample, N represents the population (N=120), Z is the normal variable, p is the true probability of the event (p=0.05), and e represents sampling error (e=0.08). The study included 103 patients with dyspepsia.

Patients were selected through convenience sampling and adherence to established eligibility criteria. The inclusion criteria were patients aged ≥18 years, of any sex, with an indication for UGE, and fasting for a minimum of eight hours and a maximum of 12 hours. Dyspepsia was defined according to the Rome IV criteria, including recurrent epigastric pain or discomfort, postprandial fullness, or early satiety lasting for at least three months, with symptom onset at least six months prior.

Patients who were pregnant or lactating, had gastric physiological disorders, such as vagotomy, prior gastric resection surgery, Zollinger-Ellison syndrome, or pyloric stenosis, and those who had undergone sleeve bariatric surgery were excluded. Patients diagnosed with anemia, hemoglobinopathies, or severe chronic diseases, and those using erythropoietin or iron supplements (factors that may interfere with the analysis of glycated hemoglobin) or lipid-lowering, hypoglycemic, antibiotic, or gastric antisecretory medications within two weeks before UGE were excluded. Data were collected from the endoscopy waiting area between October 2022 and January 2023. Patients were recruited in a pre-endoscopy waiting room after receiving information about the study objectives and methods. Those who agreed to participate signed an informed consent form and participated in the study.

Socioeconomic characteristics were collected through self-reported information during interviews, using a questionnaire covering data on sex, age, race/ethnicity, education, marital status, monthly income, smoking, and alcohol use. Data on clinical factors, endoscopic diagnoses, and *H. pylori *infection status were obtained from patient records. Bacterial detection was performed using a rapid urease test and histopathology samples derived from gastric biopsies obtained during UGE.

The patients were weighed in the waiting area in fasting conditions before UGE. Weight was measured using a digital scale, with the patients wearing light clothing, no footwear, and with an empty bladder. Height was measured with a stadiometer in a vertical position, with the patient standing against a wall, arms along the body, and head aligned to the Frankfurt plane. Body mass index (BMI) was calculated as the ratio of body weight (in kg) to the square of height in meters [20].

Body circumference was measured using a non-elastic tape with an accuracy of 0.1 cm. Neck circumference (NC) was measured with the patient seated and the tape positioned below the upper edge of the larynx, perpendicular to the neck’s longitudinal axis and below the thyroid cartilage [21]. Waist circumference (WC) was measured at the midpoint between the lower rib cage border and the iliac crest. Hip circumference was measured at the point of maximum gluteal protrusion in the horizontal plane, with the arms slightly forward and feet together. This measure was used to calculate the waist-to-hip ratio (WHR), which is defined as the ratio between the waist and hip circumferences [22]. Systolic blood pressure (SBP) and diastolic blood pressure (DBP) were measured using an aneroid device after a 10-minute rest period [23].

For analyses of fasting blood glucose and serum triglycerides, venous blood samples (15 mL) were collected using a vacuum collection system by puncture of the median antecubital vein after at least eight hours of fasting. All procedures were performed by trained professionals. Samples were divided into tubes containing fluoride for fasting glucose determination, tubes with ethylenediaminetetraacetic acid (EDTA) for glycated hemoglobin (HbA1c) analysis, and tubes with clot activator gel for serum lipid profile analysis. Analyses were performed using a CELL-DYN 1800 electronic counter (Sequoia-Turner Corporation, Mountain View, CA, USA).

Biochemical assays of glucose, triglycerides (TG), and total cholesterol (TC) were performed using enzymatic reagents and quantified photometrically with an ADVIA® 1200 autoanalyzer (Siemens Healthcare Diagnostics, Tarrytown, NY, USA). Low-density lipoprotein cholesterol (LDL-c) and very-low-density lipoprotein cholesterol (VLDL-c) fractions were calculated according to the Friedewald equation. The TyG index was calculated using the following formula [24]:

$\text{TyG index} = \ln\left( \frac{\text{triglycerides (mg/dL)} \times \text{fasting glucose (mg/dL)}}{2} \right)$

Dyslipidemia was defined as the presence of at least one of the following criteria: TC ≥ 200 mg/dL, TG ≥ 150 mg/dL, LDL-c ≥ 160 mg/dL, and high-density lipoprotein cholesterol (HDL)-c ≤ 40 mg/dL for men and ≤ 50 mg/dL for women [25].

For the analysis, dyspeptic patients were divided into four groups; Group 1: dyslipidemia (n = 27; 26.2%); Group 2: *H. pylori* infection without dyslipidemia (n = 25; 24.2%); Group 3: dyslipidemia and *H. pylori* infection (n = 12; 11.6%); Group 4: neither dyslipidemia nor *H. pylori* infection (control) (n = 39; 37.8%). The distribution of participants among the four study groups was carried out by convenience sampling, according to the confirmed diagnoses of dyslipidemia and *H. pylori* infection identified during clinical and laboratory investigation.

Continuous variables were tested for normality using the Kolmogorov-Smirnov test and assessed for homogeneity of variances with Levene’s test. Normally distributed variables are presented as mean ± standard deviation (SD) and non-normal variables as median (IQR) or range. Categorical variables are presented as counts and percentages. Between-group comparisons were performed using one-way ANOVA with Tukey post-hoc tests for normally distributed variables and Kruskal-Wallis with Dunn’s post-hoc tests for non-normal variables. Categorical variables were compared using chi-square or Fisher’s exact test as appropriate. Two-sided p < 0.05 was considered statistically significant. Effect sizes (eta squared for ANOVA or r for nonparametric tests) and 95% confidence intervals (CIs) should be reported for the main outcomes. All analyses were performed with IBM SPSS Statistics software, version 22.0 (IBM Corp., Armonk, NY, USA). Missing data were handled by complete-case analysis; if missingness exceeds 5% for a variable, perform a sensitivity analysis (e.g., multiple imputation) and report it.

Ethical considerations

The Research Ethics Committee of the Federal University of Maranhão (UFMA), Imperatriz, Brazil, issued approval (approval number: 3.212.699). 

## Results

We analyzed 103 patients with dyspeptic symptoms who attended a public endoscopy service. The sample comprised 75.2% women; the mean age was 43.8 ± 15.4 years (range 18-82 years). Regarding group distribution: Group 1: dyslipidemia (n = 27, 26.2%); Group 2: *H. pylori *infection without dyslipidemia (n = 25, 24.2%); Group 3: dyslipidemia and *H. pylori* infection (n = 12, 11.6%); Group 4: neither (control) (n = 39, 37.8%).

Upon comparing the glycemic parameters among the four groups, a statistically significant difference was observed in the TyG index values (p < 0.0001). The values varied between the groups and were higher in Group 1 and lower in Group 2.

No statistically significant differences were noted between the groups in terms of HbA1c and fasting blood glucose parameters. High HbA1c levels were found in patients in Group 3, in addition to high fasting blood glucose levels in patients in Group 1 (Table 1).

**Table 1:** Table 1. Glycemic, anthropometric parameters, and systemic blood pressure in different groups of dyspeptic patients: dyslipidemia, H. pylori infection, and control TyG: triglyceride and glucose; BMI: body mass index; NC: neck circumference; WC: waist circumference; WHR: waist-to-hip ratio; SBP: systolic blood pressure; DBP: diastolic blood pressure; *H. pylori*: *Helicobacter pylori* Data are expressed as median (range) for non-normally distributed variables and as mean ± standard deviation (± SD) for WC. *analysis of variance (ANOVA) **Kruskal-Wallis test. Values were consistently expressed according to data distribution: non-normally distributed variables (BMI and WC) are presented as median (interquartile range).

Variables	Group 1: Dyslipidemia		Group 2: *H. pylori*		Group 3: Dyslipidemia + *H. pylori*		Group 4: Control		p-value
	Median (range)	Mean ± SD	Median (range)	Mean ± SD	Median (range)	Mean ± SD	Median (range)	Mean ± SD	
TyG index	9.10 (8.56–11.5)	- -	8.18 (7.60–8.84)	- -	8.85 (8.18–9.59)	-	8.30 (7.32–10.0)	- -	<0.0001**
Glycated hemoglobin (%)	5.4 (4.1–11.3)	- -	5.1 (3.9–5.9)	- -	5.5 (4.8–8.0)	-	5.2 (4.3–12.4)	- -	0.078**
Fasting blood glucose (mg/dL)	91.0 (63.0–364.0)	- -	80.0 (66.0–100.0)	- -	81.0 (72.0–172.0)	- -	81.0 (63.5–369.5)	- -	0.078**
BMI (kg/m²)	27.2 (17.1–36.4)	- -	23.6 (20.2–40.8)	- -	30.2 (22.3–39.4)	- -	22.9 (17.0–36.0)	- -	0.007**
NC (cm)	36.0 (28.0–45.0)	- -	35.0 (24.0–39.0)	- -	37.0 (32.0-91.0)	- -	33.0 (28.0–47.0)	- -	0.05**
WC (cm)	- -	105 ± 13.5	- -	98 ± 10.9	- -	106 ± 18.3	- -	96 ± 10.1	<0.0001*
WHR	0.91 (0.66–1.32)	- -	0.88 (0.75–0.99)	- -	0.88 (0.62–1.05)	- -	0.85 (0.67–1.09)	- -	0.09**
SBP (mmHg)	130.0 (110.0–180.0)	- -	120.0 (100.0–160.0)	- -	130.0 (110.0–180.0)	- -	120.0 (90.0–180.0)	- -	0.20**
DBP (mmHg)	80.0 (60.0–110.0)	- -	80.0 (70.0–100.0)	- -	90.0 (70.0–110.0)	- -	80.0 (60.0–110.0)	- -	0.04**

Differences were observed in anthropometric parameters, such as BMI (p = 0.007), WC (p < 0.0001), and DBP (p = 0.04), among the groups of patients with dyspepsia. The highest BMI, WC, and DBP values were observed in patients in Group 3 compared to those in the other groups (Table 1).

**Figure 1:**
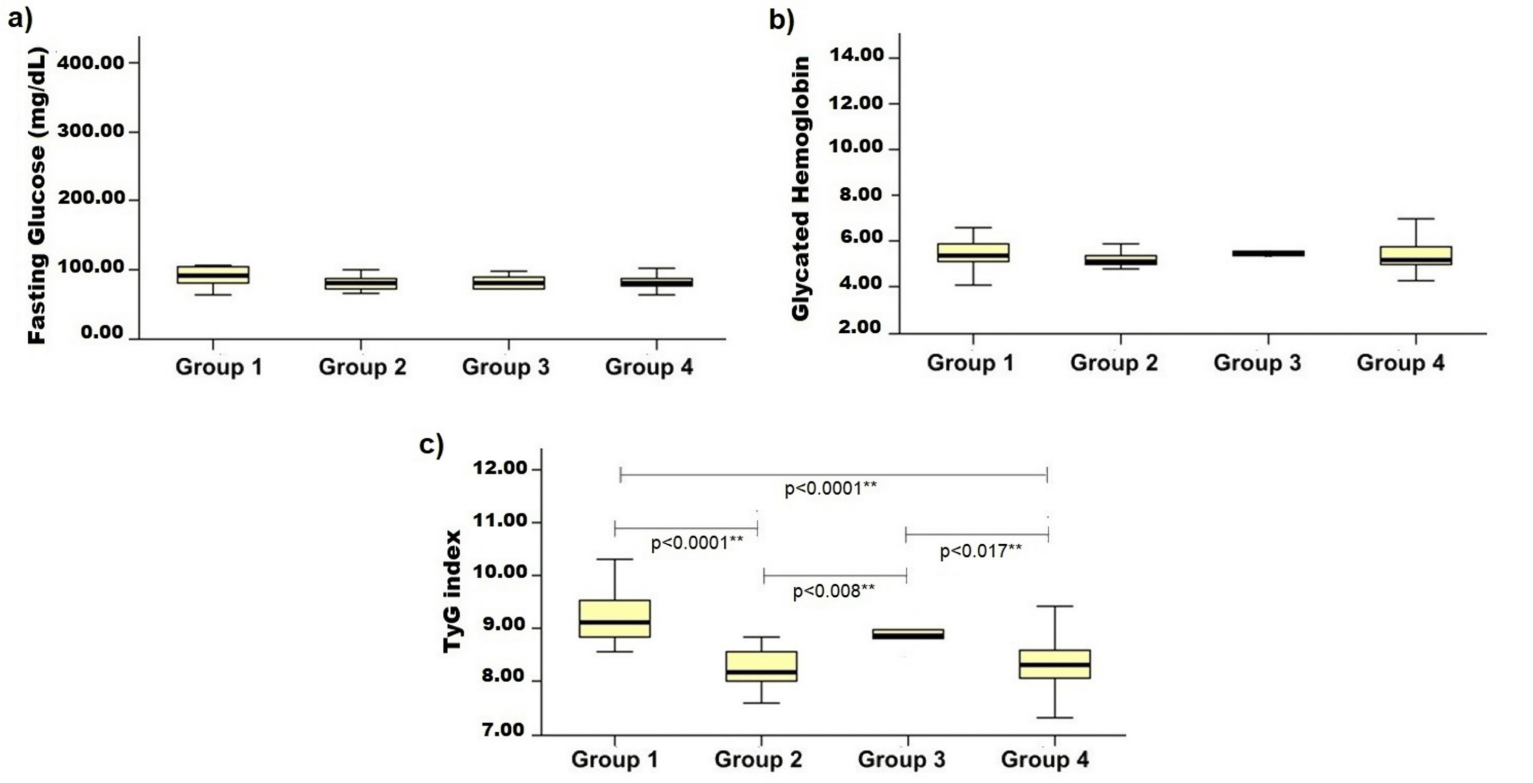
Values of a) fasting glucose, b) glycated hemoglobin, and c) TyG index in dyspeptic patient groups with dyslipidemia, H. pylori infection, and control. ** Kruskal-Wallis test with multiple comparisons-intergroup analyses (Dunn´s multiple comparison test) Group 1: dyslipidemia; Group 2: *H. pylori* infection; Group 3: dyslipidemia and *H. pylori* infection; Group 4: control TyG index:* t*riglyceride-glucose index; *H. pylori*: *Helicobacter pylori*

In the assessment of intergroup glycemic parameters, a statistically significant difference was observed in the TyG index values between patients with dyslipidemia (Group 1) and those infected with *H. pylori* without dyslipidemia (Group 2), with a higher TyG index in Group 1 and lower values in Group 2 (p < 0.0001). In addition, there was a difference in the TyG index between Group 1 and the control group (Group 4), with higher values observed in Group 1 (p < 0.0001). Significant differences were observed in the TyG index values among patients in Group 3, Group 2 (p = 0.008), and Group 4 (p = 0.017), with higher values in Group 3 (Figure 1).

Dunn’s post-hoc analysis revealed significant differences between Groups 1 and 2 (p < 0.001), Groups 1 and 4 (p < 0.001), and between Groups 3 and 2 (p = 0.008) and 3 and 4 (p = 0.017), confirming that isolated dyslipidemia and, to a lesser extent, its coexistence with *H. pylori* infection are associated with higher TyG index values.

**Figure 2:**
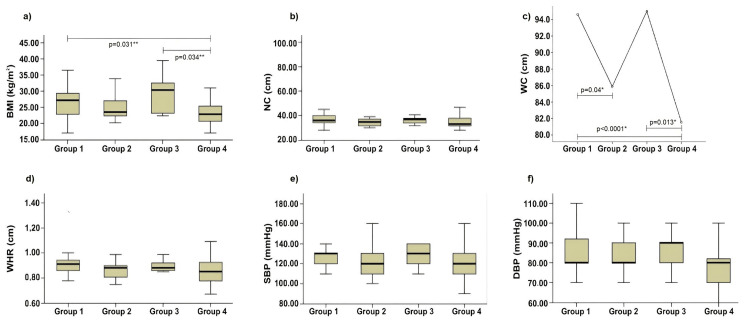
Anthropometric and blood pressure data: a) BMI; b) NC; c) WC; d) WHR; e) SBP; f) DBP in dyspeptic patients with dyslipidemia, H. pylori infection, and control. *Analysis of variance (ANOVA) with Tukey's post hoc test; ** Kruskal-Wallis test with multiple comparisons-intergroup analyses (Dunn's multiple comparison test) Group 1: dyslipidemia; Group 2: *H. pylori *infection; Group 3: dyslipidemia and *H. pylori* infection; Group 4: control BMI: body mass index; NC: neck circumference; WC: waist circumference; WHR: waist-to-hip ratio; SBP: systolic blood pressure; DBP: diastolic blood pressure; *H. pylori*: *Helicobacter pylori*

In the intergroup evaluation of anthropometric parameters, BMI values differed significantly among the healthy control group (Group 4), patients with dyspepsia and dyslipidemia (Group 1), and patients with dyspepsia, dyslipidemia, and *H. pylori* infection (Group 3), with higher BMI values in Group 3. Significant differences in WC were observed between the healthy control group (Group 4) and Groups 1 and 3, with higher WC values observed in patients in Group 3. In addition, a difference was observed between Groups 1 and 2, with higher WC values in Group 1 (Figure 2).

## Discussion

The TyG index is a marker of IR that reflects lipid and glucose metabolism. TyG index thresholds >8.5 have been associated with elevated risks of cardiovascular disease and mortality in adults [26]. This difference was confirmed by post-hoc comparisons showing significantly higher TyG indices in patients with isolated dyslipidemia and in those with coexisting dyslipidemia and *H. pylori* infection compared with the other groups. Here, patients with dyslipidemia and without *H. pylori* infection had median TyG indices of 9.1, which were higher than those found in infected patients without dyslipidemia, with median values ​​of 8.1. This difference was confirmed by post-hoc comparisons showing significantly higher TyG indices in patients with isolated dyslipidemia and in those with coexisting dyslipidemia and* H. pylori* infection compared with the other groups.

Furthermore, no significant differences were observed in HbA1c and fasting glucose levels between the groups. However, median HbA1c values ​​were higher in patients with dyslipidemia who were infected with *H. pylori*, while median fasting glucose values ​​were higher in patients with dyslipidemia who were not infected. This may indicate that, although dyslipidemia and *H. pylori *infection influence lipid metabolism, their direct effect on glycemic control is less pronounced in patients with dyspepsia without established diabetes. Our sample, which consisted primarily of individuals without diabetes, may explain the lack of significant variation in these markers.

Previous studies have not identified significant differences in blood glucose levels based on the presence of *H. pylori* infection between individuals with and without diabetes. Furthermore, eradication of the bacteria had no significant effect on fasting blood glucose and IR [16, 17]. Similarly, in a study of over 37,000 individuals, *H. pylori* infection was associated only with dyslipidemia, with no significant relationship to fasting blood glucose or HbA1c levels [27].

However, some studies have demonstrated an association between the presence of *H. pylori* and glycemic control in individuals with diabetes, suggesting that eradication of the bacteria could benefit glycemic control [7, 8, 28]. In the present study, patients with *H. pylori* infection without dyslipidemia had lower TyG index values, indicating that infection alone may not be sufficient to induce IR. These findings contrast with studies that associate the presence of *H. pylori *infection with an increased risk of metabolic syndrome [6, 29] and elevated TyG indices [14, 15], but reinforce the relevance of considering concomitant factors, such as dyslipidemia and adiposity, in the development of metabolic disorders. In the present study, patients with dyslipidemia and *H. pylori* infection had high BMI and WC, indicating that the coexistence of these conditions may be associated with a more obesogenic profile. This corroborates evidence that *H. pylori* infection is associated with changes in body composition and visceral fat accumulation [5, 11, 16]. Bacteria-induced chronic inflammation can interfere with appetite regulation and energy metabolism, worsening metabolic risk in individuals already predisposed to dyslipidemia.

One study showed that elevated BMI indices were associated with *H. pylori* infection; however, no significant association was observed between *H. pylori* infection and triglyceride, LDL-c, or HDL-c levels [19]. Another study that analyzed changes in metabolic parameters after *H. pylori* eradication showed that HDL-c levels and BMI increased one year after eradication therapy and varied by sex, with significantly higher HDL-c levels in women and higher BMI in men [30]. *H. pylori* infection has been suggested to increase serum leptin secretion, which delays satiety and increases food intake, thus contributing to weight gain [11].

The heterogeneity of the populations evaluated and variations in diagnostic criteria may contribute to discrepancies in study results, reinforcing the need for additional longitudinal research.

In terms of clinical implications, these results suggest that screening for dyslipidemia in dyspeptic patients (and vice versa) can identify individuals at higher cardiometabolic risk in settings where routine endoscopy is performed. The TyG index, an inexpensive and easily obtained marker from glucose and triglyceride measurements, may be useful for early risk stratification, aiding in the prevention of cardiovascular disease. These findings offer valuable guidance for future research exploring the diverse mechanisms by which *H. pylori* infection influences lipid metabolism and body fat distribution.

This study has some limitations. Patients' dietary habits and inflammatory biomarkers were not assessed. Because this was a cross-sectional study, patient follow-up was not possible, and the sample size was limited to a single endoscopy service.

## Conclusions

Our results showed that patients with dyspepsia, dyslipidemia, and without *H. pylor*i infection had greater IR, evidenced by higher TyG indices, compared to those with isolated *H. pylori* infection. However, the coexistence of dyslipidemia and *H. pylori* infection led to excess body mass and visceral fat accumulation, as evidenced by high BMI and WC. These findings suggest that dyslipidemia exerts a greater influence on IR than isolated *H. pylori* infection, while their coexistence is associated with increased central adiposity. Large-scale longitudinal studies are warranted to confirm these associations and to elucidate the pathophysiological mechanisms linking metabolic dysfunction and chronic gastrointestinal infection.
